# Prevalence of anal human papillomavirus infection and cytologic abnormalities among HIV-infected and HIV-uninfected men who have sex with men

**DOI:** 10.7448/IAS.17.4.19662

**Published:** 2014-11-02

**Authors:** Alessandra Latini, Maria Gabriella Dona, Livia Ronchetti, Amalia Giglio, Domenico Moretto, Manuela Colafigli, Valentina Laquintana, Mirko Frasca, Mauro Zaccarelli, Andrea Antinori, Antonio Cristaudo, Massimo Giuliani

**Affiliations:** 1 Infectious Dermatology and Allergology Unit, San Gallicano Dermatologic Institute, IRCCS, Rome, Italy; 2 Pathology Department, Regina Elena National Cancer Institute, IRCCS, Rome, Italy; 3 Clinical Pathology and Microbiology DepartmentSan Gallicano Dermatologic Institute, IRCCS, Rome, Italy; 4 Clinical Department, National Institute for Infectious Diseases “Lazzaro Spallanzani”, Rome, Italy

## Abstract

**Introduction:**

Human papillomavirus (HPV) is responsible for 85% of anal cancers. Recently, anal cancer incidence has been increasing, particularly in men who have sex with men (MSM). Cytology may be a useful tool for the detection of anal precancerous lesions. We assessed the prevalence and determinants of anal HPV infection and cytologic abnormalities among HIV-infected and -uninfected MSM.

**Materials and Methods:**

MSM ≥18-year-old attending an STI clinic in Rome (Italy) were enrolled. Anal cytologic samples were collected in PreservCyt (Hologic) using a Dacron swab. The Linear Array HPV Genotyping Test (Roche Diagnostics) was used for the detection and genotyping of 37 mucosal HPV types. Liquid-based cytological slides were obtained using a ThinPrep2000 processor (Hologic). The morphology of the anal pap-test was classified following the Bethesda 2001 guidelines.

**Results:**

We enrolled 180 HIV-infected (median age 41 years, IQR 33–47) and 438 HIV-uninfected MSM (median age 32 years, IQR: 27–39). Most of the individuals were Caucasian (92.2% and 97.0%, respectively). HPV prevalence, both overall (93.3% vs 72.4%, p<.001) and by high-risk (HR) HPV types (80.5% vs 56.0%, p<.001), was significantly higher among HIV-infected than HIV-uninfected individuals. HPV-multiple infections were evidenced in 48.2% of the HIV-uninfected and 76.1% of the HIV-infected MSM (p<.001). HPV16 was the most prevalent genotype in both groups (23.3% in HIV-positive and 17.6% in HIV-negative MSM). HPV6 and 84 were the most frequent low-risk types in both cohorts. Anal cytologic abnormalities were found in a significantly higher proportion of HIV-infected MSM (46.1% vs 27.9%, p<.001). H-SILs (high-grade squamous intraepithelial lesions) were exclusively observed among the HIV-infected individuals, although at a low prevalence (1.2%).

**Conclusions:**

A high prevalence of anal HPV infection and cytologic abnormalities was evidenced in both populations. Nonetheless, HIV-infected MSM showed a significantly higher rate of HPV infection and abnormal cytology, confirming that HIV-1 infection poses a significant risk for anal HPV infection as well as for anal cellular abnormalities. Screening for anal cancer, which is currently the most frequent non-AIDS-defining cancer in HIV-positive MSM, should be considered for this population. Moreover, vaccination strategies for the prevention of HPV infection should be taken into account.

